# Observational Evidence of For-Profit Delivery and Inferior Nursing Home Care: When Is There Enough Evidence for Policy Change?

**DOI:** 10.1371/journal.pmed.1001995

**Published:** 2016-04-19

**Authors:** Lisa A. Ronald, Margaret J. McGregor, Charlene Harrington, Allyson Pollock, Joel Lexchin

**Affiliations:** 1 Department of Family Practice, University of British Columbia, Vancouver, British Columbia, Canada; 2 School of Nursing, University of California, San Francisco, San Francisco, California, United States of America; 3 Queen Mary, University of London, London, United Kingdom; 4 Centre for Primary Care and Public Health, Blizard Institute, Barts and The London School of Medicine and Dentistry, London, United Kingdom; 5 School of Health Policy and Management at York University, Toronto, Ontario, Canada; 6 Department of Family and Community Medicine, University of Toronto, Toronto, Ontario, Canada

## Abstract

Margaret McGregor and colleagues consider Bradford Hill’s framework for examining causation in observational research for the association between nursing home care quality and for-profit ownership.

**Summary Points**

- Nursing home residents are a highly vulnerable population, and nursing home care quality has been a persistent focus of public concern.
- There is considerable evidence from observational studies that public funding of care delivered in for-profit facilities is inferior to care delivered in public or nonprofit facilities.
- The past decade has seen many industrialized countries increasing governmental payment for care of frail seniors in for-profit nursing homes, leading to questions about whether this leads to inferior care.
- Many of Bradford Hill’s guidelines for causation can be found in published studies supporting a causal link between for-profit ownership and inferior care.
- The precautionary principle should be applied when developing policy for this frail and vulnerable population.

## Introduction

Nursing homes, also called residential long-term care facilities or aged care homes, are regulated institutions providing around-the-clock medical and social care to (mainly) older people who are unable to live independently due to physical and/or mental disability. Because of the vulnerability of this population and frequent media reports of scandals across many industrialized countries [1], nursing home care quality has been a persistent focus of public concern.

Inserted into the discourse on quality has been a trend in many countries to contract care to for-profit–owned facilities, and there has been considerable effort by researchers to understand the impact of for-profit ownership on care quality. Research into this area is not new [2]. O’Brien and colleagues asked the question in a 1983 review in which they included several United States studies going back to 1968 [3]. However, recent examples such as the 2011 failure of the largest United Kingdom private equity nursing home chain, Southern Cross [4], and a report in 2000 that the five largest US nursing home chains operated under bankruptcy protection [5], have brought this policy question to the forefront.

The issue has particular relevance at this time as jurisdictions are challenged to care for an increasing number of very frail people over the next two decades [6]. Even with policies to expand care at home, it is likely that many countries will require the construction of new nursing home beds [7].

In this paper, we evaluate the evidence for an association between for-profit ownership and inferior care, using Bradford Hill’s framework for examining causation in observational research. We further frame the issue in terms of the precautionary principle, asking, “At what point is it is time to shift policy direction based on the available evidence?”

## Trends in Nursing Home Ownership and Care Delivery in Industrialized Countries

Nursing homes can be owned and operated by public (government or quasi-governmental), nonprofit, or for-profit entities, with differences among countries in financing, regulation, and mix of ownership. Box 1 describes a taxonomy of different ownership types. Regardless of the ownership and delivery of nursing home care, the majority of funding for nursing home care in industrialized countries comes from public sources [8].

**Box 1. Taxonomy of Nursing Home Ownership**

**Public ownership:** facilities owned by government or quasi-governmental bodies. Municipal governments, health regions, and Veterans Affairs would be examples of public and quasi-public owners.

**Nonprofit ownership:** nongovernmental ownership by religious or community groups or agencies, in which the facilities they operate are run as nonprofit societies. A nonprofit society or entity is constituted with the assumption that any revenue in excess of expenses will be used to benefit its clients.

**For-profit facilities:** owned and operated as businesses. Here it is assumed that revenue in excess of expenses can be directed to the owners—or, in the case of shareholder-owned companies, to shareholders. They include both small provider-owned facilities and large corporate chains whose headquarters are not necessarily in the province, or even the country, where they operate. The distinction between provider ownership and corporate ownership can be important: when facility owners are also care providers, it is fair to expect that—at least theoretically—their professional obligation balances the sometimes conflicting motivations of generating profit and providing good-quality care.

Table 1 summarizes trends in nursing home ownership across a range of years and countries. In the UK in 2012, 78% of residential care beds were in for-profit–owned facilities, a 10% increase from 2007 [9]. In Australia, roughly one-third of nursing home beds are owned by private for-profit companies [10]. In New Zealand, a survey of New Zealand Care Association members reported that approximately two-thirds of nursing homes were for-profit, a trend that was increasing [11]. In the US, more than two-thirds of beds are for-profit, with more than half owned by corporate chains [12,13].

**Table 1:** Percent of nursing home beds, by for-profit and nonprofit ownership

Country	Year	For-profit ownership	Public and nonprofit ownership
United Kingdom [9]	2012	78%	22%^†^
	2007	74%	26%
New Zealand [11]	2009	76%	24%
	2005	65%	35%
USA [12]*	2008	67%	33%^‡^
	2003	66%	34%
Canada [14]	2011	37%	63%
	2008	35%	65%
Australia [10]*	2007	27%	73%^§^
Sweden [15]	2012	21%^#^	79%
	1993	5%^#^	95%

Note: For-profit–owned facilities include both publicly and privately funded delivery of services.

* Percent of nursing homes reported

^†^ 8% government-owned, 14% private nonprofit

^‡^ 6% government-owned, 27% private nonprofit

^§^ 12% government-owned, 61% private nonprofit

^#^ Reported value includes both private for-profit and private nonprofit ownership. However, as stated by the authors, “The entire increase of private provision is the result of the growth of for-profit—in contrast to nonprofit—providers” (p. 23, [15]).

The past decade has also seen the movement of private equity and other investor-owned firms into the nursing home sector, in both the US and other industrialized countries [4,13,16]. Some have termed this a “caretelization” of the nursing home industry, whereby large corporate providers have gained greater market share through the process of mergers, acquisitions, and takeovers [4].

## Evaluating the Observational Evidence Using Bradford Hill’s Guidelines

There are challenges to measuring care quality (Box 2). Nonetheless, three systematic reviews have concluded that for-profit nursing homes had poorer care quality than nonprofit-owned homes [17–19]. A large meta-analysis found that two of four outcomes were significantly superior in nonprofit compared to for-profit homes [17]: more or higher quality staffing (ratio of effect 1.11, 95% confidence interval [CI]: 1.07 to 1.14), and lower pressure ulcer prevalence (0.91, 95% CI: 0.83 to 0.98). Non-significant results were found for the two other outcomes: fewer deficiencies in governmental regulatory assessments (0.90, 95% CI: 0.78 to 1.04) and lower physical restraint use (0.93, 95% CI: 0.82 to 1.05). The authors estimated that residents would receive 42,000 and 500,000 additional hours of nursing care per year, and have 600 and 7,000 fewer pressure ulcers in Canada and the US, respectively, if these services were provided solely by nonprofit facilities [17]. In 40 of the 82 studies reviewed, all statistically significant measures of quality favored nonprofit facilities, compared to only three studies in which all measures favored for-profit facilities [17].

**Box 2. How Is Care Quality Measured in Nursing Homes?**

Measurement of care quality in nursing homes is multidimensional, with numerous definitions, a vast range of indicators, and no gold standard for measurement [20,21]. Examples of different quality indicators include structural (such as staffing levels and training), process (such as inspection violations, continuity of care, prevalence of daily physical restraints, and indwelling catheters), and outcome indicators (such as prevalence of pressure sores, urinary tract infections, avoidable hospital admissions and dehydration) [20]. There is also growing recognition that, beyond staffing measures, there has been little progress in measuring resident- and family-reported experience of care [22,23], which is arguably one of the most meaningful measures in this population [23].

While no single indicator represents the overall quality of a nursing home, a disadvantage of using multiple quality indicators is that findings can be inconsistent [24]. A detection bias can also occur whereby rates of adverse outcomes may be higher in nursing homes or jurisdictions that actively “look for” problems [24]. Small numbers of events and small average facility size can limit the power of statistical analyses to find an effect [24]. This can lead to wide confidence intervals around estimates and conclusions that observed trends are not statistically significant [24]. Confounding can also result when comparing indicators between facilities, since patient case mix can vary between facilities [24]. Finally, many nursing homes are measured on self-reported indicators, leading to potential reporting bias for some indicators.

An editorial accompanying the above-described meta-analysis implied that the observational evidence is too weak for policy decisions, and that because of the impossibility of conducting randomized controlled trials of profit versus nonprofit status, causation cannot be proven [25]. This brings us to a theoretical debate about how we determine a link is causal when all we have, and all we are ever likely to have, is evidence from observational studies.

We use the Bradford Hill framework to assess whether there is sufficient evidence to suggest causation [26]: the presence of plausibility, temporality, experiment, dose-response, coherence, analogy, consistency, magnitude of effect, and specificity (Box 3):

**Box 3. Bradford Hill’s Guidelines for Assessing Causation**

It is usually accepted that high-quality randomized controlled trials (RCTs) are able to overcome bias and confounding and, therefore, top the evidence hierarchy to provide sufficient evidence to establish a causal link between exposure and outcome [27]. However, properly conducted RCTs in many areas are rare—trials can be underpowered, unsuccessfully blinded, and suffer from undetected biases [27]. Furthermore, not all research questions can be investigated using RCTs. In the case of nursing homes, it would be neither ethical nor feasible to randomly assign facility ownership or care delivery to for-profit versus public or nonprofit status. Thus, we rely on observational studies to evaluate the relationships between quality of care and ownership, in which we observe rather than assign exposures. Criticisms of observational studies, however, are that they are more prone to bias and confounding.

Some suggest that guidelines for causation can be a useful tool for assessing if there is sufficient evidence before concluding causation [27]. The British epidemiologist, Sir Austin Bradford Hill, developed guidelines to evaluate evidence for a causal effect [26]. These guidelines, first published in 1965, in part to address the link between tobacco and lung disease, provide a useful framework for assessing evidence for a causal effect. Specifically, Bradford Hill suggested that nine relevant factors should be considered before concluding causation [26]:

**Plausibility:** The cause-and-effect interpretation of an association should fit with the known facts of the natural history and biology of the disease.

**Temporality:** A necessary criterion for a causal association is that the exposure must precede the outcome.

**Experiment:** Causation is more likely if evidence is based on randomized experiments.

**Biological gradient or dose-response:** The likelihood of a causal association is increased if a dose-response curve can be demonstrated.

**Coherence:** A causal conclusion should not contradict present substantive knowledge.

**Analogy:** For analogous exposures and outcomes, an effect has already been shown.

**Consistency:** A relationship is observed repeatedly, prospectively and retrospectively, in different populations.

**Strength of the association:** Strong associations are more likely to be causal than weak associations.

**Specificity:** If an association is limited to specific groups with a particular environmental exposure or is greatly increased in these groups, then the case for a causal association is strengthened.

### Plausibility

All nursing homes must balance their revenues and expenses in order to survive. For-profit organizations operate on the principle that profits or net income (revenue in excess of expenses) is directed to the owners, investors, or shareholders [28]. In nonprofit organizations and publicly owned facilities, net income is used to benefit clients [28].

O’Neill describes the trade-off between profit and quality: “If increasing quality raises costs more quickly than it does revenues, profits must fall as quality improves” [29]. In order to generate profits, for-profit homes tend to have lower costs and lower staff-to-patient ratios than nonprofit facilities [30]. Money diverted to shareholders and investors leaves less money to pay for staff, and in turn, having fewer or untrained staff is associated with lower quality [31–34].

The lower level of staffing with for-profit ownership [17,18] stands in contrast to the well-established association between higher levels of total nursing and registered nursing staff and better care outcomes [31–34]. Nurse staffing levels have a positive impact on both the process and the outcomes of nursing home care, such as reduced resident time in bed, improved feeding assistance, incontinence care, exercise and repositioning [33], fewer regulatory deficiencies [35], and lower rates of pressure ulcers [17]. Higher staffing levels are associated with lower staff turnover [36]—a pre-condition for good relational care, which in turn is associated with improved quality of life [23] (i.e., relational care embraces the entire relationship between caregiver and care recipient, encompassing the physical, social, emotional, and spiritual dimensions of human connection [37,38]). In a US study, the largest ten for-profit chains had lower registered nurse and total nurse staffing hours and a 41% higher number of serious deficiencies than government facilities, controlling for other factors [30].

A second plausible mechanism proposed for the “for-profit” effect of inferior outcomes is that for-profit facilities have a lower threshold for transferring acutely ill residents to acute care facilities [39–42]. This higher rate of use of acute services (emergency department visits and hospital admissions) among residents in for-profit facilities has been a consistent finding and is thought to be in part related to avoidance of the higher costs associated with caring for acutely ill residents [39–42]. Hospital admission for nursing home residents is considered a poor outcome because it puts these residents at risk of iatrogenic infections [43], falls, delirium, and decline in functional status and quality of life [44]. Furthermore, there is now some evidence that illnesses such as pneumonia can be equally well managed within the facility [45].

A third plausible mechanism for the association of nonprofit and/or public facilities with improved quality of care may be related to their ability to become charitable foundations. In many jurisdictions, this status provides tax breaks and makes them better positioned to mobilize volunteers and solicit donations for equipment [46].

In ideal market conditions, residents’ should be able to “exit” (leaving the facility) or use “voice” (complaining) [47]. However, the high degree of vulnerability of the nursing home population and the information asymmetry required for meaningful choice make these ineffective as counterbalances to behaviors that sacrifice quality [48,49].

### Temporality

Temporality has been investigated in several studies by examining conversions between ownership types. Longitudinal observational research from the US [50] and Sweden [51] has found that nursing homes converting to for-profit ownership demonstrated a subsequent decline in some quality measures. Nursing homes converting from for-profit to nonprofit status generally exhibit improvement both before and after conversion [52]. A major challenge to such research is the potentially confounding effect of unmeasured differences in nursing homes that choose to convert [50] compared to those who do not.

### Experiment

While it is unlikely that experimental evidence from randomized trials will ever be available to compare nursing home ownership and quality, two US studies [39,40] have recently used a method (instrumental variables analysis) that mimics randomization. This approach can estimate causal relationships when it is not possible to conduct a randomized trial.

The two studies examined a national cohort of newly admitted residents to short- [39] and long-stay facilities [40], including almost 14,000 US nursing homes. Data were drawn from national standardized clinical data (Minimum Data Set, MDS) linked to Medicare claims over an 18-month period between 2004 and 2005. Authors mimicked randomization of residents into more or less “exposure” to nonprofit homes by using “differential distance” to the nearest nonprofit nursing home relative to the nearest for-profit nursing home. Both studies found higher rates of hospital admissions and one study [39] demonstrated inferior outcomes for mobility, pain, and function measures among residents living in for-profit facilities compared to nonprofit facilities. The authors concluded that the observed effects were likely causal and could not be explained by unmeasured differences in case mix between facilities with different ownership structures.

### Dose-Response Effect

A gradient effect between profit margins and US nursing home inspection violations has been reported [29]. O’Neill and colleagues examined 952 for-profit facilities in California to assess the relationship between profit and the number of total and serious deficiencies reported by regulatory inspectors. Authors divided facilities into four profit categories from the lowest to the highest profit group. After controlling for resident case mix and other facility and market characteristics, the authors found the highest profit group had significantly more total deficiencies than those in the second-highest profit group. They also found that facilities in the highest profit group had significantly more serious deficiencies than the three lower profit groups, suggesting an inverse gradient (dose-response) effect of profit on quality [29].

### Coherence, Analogy, and Consistency

Parallel studies have found for-profit services in sectors other than residential long-term care to be of inferior quality, including hemodialysis centers [53] and Health Maintenance Organizations (HMOs) [54]. Outside of the health sector, studies looking at the daycare sector in Canada [55,56] have found a similar quality gap between for-profit and nonprofit ownership.

The majority of studies evaluating nursing home ownership and care quality have used US data [17], where the distinction is typically between private for-profit and nonprofit. Studies have also reported the association between for-profit status and inferior care when compared to either nonprofit or public models in other countries, including Canada [57–60], Israel [61], and Australia [62]. While most studies are from industrialized, high-income countries, we find no reason to expect that evidence from low- and middle-income countries would be different.

### Strength of the Association

The differences reported in observational studies associated with for-profit status have generally not been large (with reported relative risks between 1 and 2) [17]. However, the magnitudes of effect are often small in studies of health care interventions, reflecting the implementation of interventions within complex systems [63].

### Specificity

This term refers to the causative agent resulting in very specific effects. This criterion is more relevant to a biomedical (versus a health systems or policy) paradigm—for example, the assumption that mesothelioma, a very specific type of lung cancer, is only seen when an individual has been exposed to asbestos [64]. While the concept is of limited application in the health policy arena, the strongest empirical evidence exists for the association of for-profit status and lower staffing levels. Since the number of staff hired is also the most costly line item with the greatest likelihood of affecting profit, one might argue that there is some degree of specificity to the association.

## Nursing Homes Are Complex Adaptive Systems and Context “Matters”

Nursing homes are complex adaptive systems [65], and health policy research, unlike biomedical research, is unlikely to discover one causal link to any system-level outcome. The association between for-profit ownership and inferior care is not a simple one.

In predominantly for-profit environments, some not-for-profit groups, despite their mandate, operate more as competitive market entities, with the focus often shifting towards increasing revenues at the expense of quality. Conversely, in jurisdictions dominated by the nonprofit or public sector, overall quality for the whole region is generally found to be better, including care delivered in for-profit nursing homes [66]. One interpretation is that the predominantly public sector raises the bar for all facilities, thus mitigating the effect of profit-making on quality. Such findings, rather than refuting the plausibility of the observed association, speak to various predisposing and mitigating contextual factors.

Additionally, where comparisons of quality have subdivided nonprofit ownership into governmental (publicly owned) and nonprofit groups, there is often a hierarchy of outcomes, whereby public models are superior to both for-profit and nonprofit models and for-profit models are inferior to public and nonprofit owned organizations [58,67,68].

## When Is There Sufficient Evidence for Policy Change?

Bradford Hill did not prescribe these guidelines as rules that must be fulfilled before an association can be judged as causal, but as a way of examining if cause and effect is the reasonable inference [69]. In the current case, some of the Bradford Hill criteria are clearly met, while others are less clear.

At the very least, the precautionary principle should apply to this highly vulnerable nursing home population. The precautionary principle shifts the debate by calling for preventive action, even when there is uncertainty but credible evidence of potentially significant impacts. This shift in burden of proof is based on the obvious premise that harms to the public's health should be avoided and that society should not have to wait for conclusive evidence before acting to protect itself [70]. Taking a precautionary approach emphasizes our responsibility to ask, when do we know enough to act as if something is causal? [71]

## What Are the Policy Challenges?

The policy response to the evidence on facility ownership clearly depends on jurisdictional context. In jurisdictions contemplating construction of new nursing home beds, policy makers need to support public and nonprofit facility ownership. Possible policy approaches include the sale of government savings bonds to raise public funds for capital construction [72], providing support to nonprofit societies with the necessary expertise for them to make competitive bids on requests for proposals, and valuing social capital and links with the community in the bidding process [7].

All jurisdictions should require public funding be earmarked and spent on mandated minimum direct care staffing levels consistent with the evidence, with no discretion for facilities to re-direct this money to other budgetary items (including profit generation). In countries where a majority of facilities are owned by large for-profit chains, proposed “downstream” policy approaches include improved financial transparency of how public resources are spent and the adoption of cost controls on administration [73]. Unfortunately, these approaches are costly to implement [74].

Decision-makers have a responsibility to ensure nursing home public policy is most consistent with the available evidence and least likely to cause harm. The majority of funding to operate and deliver care in nursing homes is derived from public, taxpayer-funded sources. When provided by the for-profit sector, the evidence suggests there is a greater likelihood of inferior care. It is time to re-align policy with evidence. Our seniors deserve better.

## Supporting Information

S1 Text Summary Points in Chinese. (PDF)

S2 Text Summary Points in French. (PDF)

## Abbreviations

CI: confidence interval
HMOs: Health Maintenance Organizations
MDS: Minimum Data Set
RCTs: randomized controlled trials
