# Supplementary material for: Observational Evidence of For-Profit Delivery and Inferior Nursing Home Care: When Is There Enough Evidence for Policy Change?
Source: PLoS Med. 2016 Apr 19;13(4):e1001995. doi: 10.1371/journal.pmed.1001995 (PMC4836753; doi:10.1371/journal.pmed.1001995)
Supplement: S1 Text — (PDF) [file pmed.1001995.s001.pdf]

要点

入住养老院的人是高危弱势群体，养老院的护理质量一直是公共关注的焦点

通过观察研究所得到的大量证据显示，同样是公共资金，用于盈利性养老机构其所提供的护理质量低于用于非盈利性机构其所提供的护理质量。

过去十年中，许多工业化国家政府增加了对盈利性养老机构护理资金的投入，引发了对此举是否导致护理质量下降的众多质疑

已经发表的研究中，很多布拉德福德•希尔（Bradford Hill）的因果关系理论证明盈利体制与低劣的护理服务质量之间存在因果关系

在为这些弱势群体制定政策时，必须运用预防原则
