# Supplementary material for: Observational Evidence of For-Profit Delivery and Inferior Nursing Home Care: When Is There Enough Evidence for Policy Change?
Source: PLoS Med. 2016 Apr 19;13(4):e1001995. doi: 10.1371/journal.pmed.1001995 (PMC4836753; doi:10.1371/journal.pmed.1001995)
Supplement: S2 Text — (PDF) [file pmed.1001995.s002.pdf]

Messages clés

- Les pensionnaires des foyers de soins infirmiers constituent une population extrêmement vulnérable et la qualité des soins qui y sont offerts préoccupe le public depuis longtemps.
- De nombreuses études d'observation montrent qu'avec un financement public égal, la qualité des soins prodigués dans les établissements à but lucratif est inférieure à celle des soins offerts dans les établissements publics ou à but lucratif.
- Au cours de la dernière décennie, beaucoup de pays industrialisés ont augmenté le financement public destiné aux soins offerts aux personnes âgées fragiles dans les établissements à but lucratif, et certains se demandent si cela fait diminuer la qualité des soins.
- Bon nombre des lignes directrices de Bradford Hill en matière de causalité se retrouvent dans des études publiées qui appuient le lien de causalité entre propriété à but lucratif et qualité inférieure des soins.
- Il faut appliquer le principe de précaution dans l'élaboration de politiques destinées à cette population fragile et vulnérable.
